# Physics-Guided Hierarchical Neural Networks for Maxwell’s Equations in Plasmonic Metamaterials

**DOI:** 10.1021/acsphotonics.5c00552

**Published:** 2025-07-31

**Authors:** Sean Lynch, Jacob LaMountain, Bo Fan, Jie Bu, Amogh Raju, Daniel Wasserman, Anuj Karpatne, Viktor A. Podolskiy

**Affiliations:** † Miner School of Computer Science, 14710 University of Massachusetts Lowell, Lowell, Massachusetts 01854, United States; ‡ Department of Physics and Applied Physics, 14710 University of Massachusetts Lowell, Lowell, Massachusetts 01854, United States; § Department of Computer Science, Virginia Tech, Blacksburg, Virginia 24061, United States; ∥ Department of Electrical and Computer Engineering, 12330 University of Texas Austin, Austin, Texas 78712, United States

**Keywords:** machine learning, metamaterials, plasmonics, physics-guided machine learning

## Abstract

While machine learning (ML) has found multiple applications in photonics, traditional “black box” ML models typically require prohibitively large training data sets. Generation of such data, as well as the training processes themselves, consume significant resources, often limiting practical applications of ML. Here, we demonstrate that embedding Maxwell’s equations into ML design and training significantly reduces the required amount of data and improves the physics-consistency and generalizability of ML models, opening the road to practical ML tools that do not need extremely large training sets. The proposed physics-guided machine learning (PGML) approach is illustrated on the example of predicting complex field distributions within hyperbolic meta­material photonic funnels, based on multilayered plasmonic–dielectric composites. The hierarchical network design used in this study enables knowledge transfer and points to the emergence of effective medium theories within neural networks.

## Graphical abstract

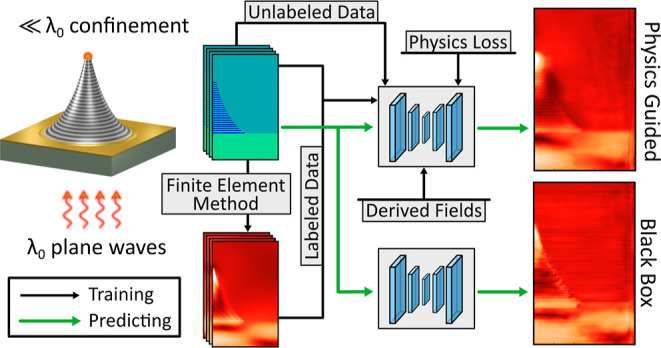

## 1 Introduction

Composite materials with engineered optical properties, metamaterials and metasurfaces, are rapidly advancing as platforms for optical communications, sensing, imaging, and computing.^1–7^ The complexity of typical metamaterials makes it almost impossible to understand and optimize their interaction with light based on experimental or analytical theory approaches alone, leaving the problem of light interaction with metamaterials to computational sciences.^1,4,8,9^ Currently, finite-difference time domain (FDTD)^10^ and finite element methods (FEM)^11,12^ represent industry-standard approaches to understanding the optics of nonperiodic composite media.

Machine learning (ML) techniques, particularly neural networks (NNs), have recently been incorporated into the design, evaluation, and measurement of nanophotonic structures.^13–21^ Properly trained ML tools can be used as surrogate models that predict the spectral response of composites or—rarely—field distributions within metamaterials.^14,22–24^ Since ML does not solve the underlying electromagnetic problem, these predictions are significantly faster than brute-force simulations. However, extensive training sets, often featuring ∼10^3^ to 10^5^ configurations are required in order to develop high-quality ML models.^14,25–28^ The time and computational resources needed to generate these data sets, as well as the time and resources needed for the ML training process, are significant and often serve as the main limitation to ML use in computational photonics.

Embedding physics-based constraints (physics-consistency)^29,30^ into the ML training process may be beneficial for the resulting models. ML methods for general solutions of partial differential equations (PDEs) are being developed.^31–38^ However, as of now, these techniques are illustrated on convenient “toy” models and cannot be straightforwardly applied to practical electromagnetic problems. Physics-guided machine learning (PGML)^6,7,39^ is emerging as a promising platform that can combine data- and physics-driven learning. Notably, previous PGML attempts have been focused on dielectric^39^ or relatively simple plasmonic^7^ composites. Here, we develop PGML models that are capable of predicting electromagnetic fields within plasmonic metamaterials. We illustrate our technique by analyzing the optical response of metamaterials-based photonic funnels:^40–42^ conical structures with strongly anisotropic composite cores that are capable of concentrating light to deep subwavelength areas. We show that physics-based constraints enable training on unlabeled data and significantly improve the accuracy and generalizability of the models. We also attempt to understand the inner workings of the NNs by analyzing the performance of hierarchical models with different data resolutions.

## 2 Hyperbolic Metamaterial-Based Photonic Funnels

An electromagnetic composite comprising sufficiently thin alternating layers of nonmagnetic materials with permittivities ϵ_1_, ϵ_2_ and thicknesses *d*_1_, *d*_2_ (see Figure 1) behaves as a uniaxial medium whose optical axis is perpendicular to the layers (direction *ẑ* in this work) and whose diagonal permittivity tensor has components given by $\epsilon_{xx}=\epsilon_{yy}=\epsilon_{⊥}=\frac{d_{1}\epsilon_{1}+d_{2}\epsilon_{2}}{d_{1}+d_{2}}$ and $\epsilon_{zz}=\frac{\left(d_{1}+d_{2}\right)\epsilon_{1}\epsilon_{2}}{d_{1}\epsilon_{2}+d_{2}\epsilon_{1}}$ . Such a material supports the propagation of two types of plane waves that differ in their polarization and have fundamentally different dispersions.

**Figure 1:**
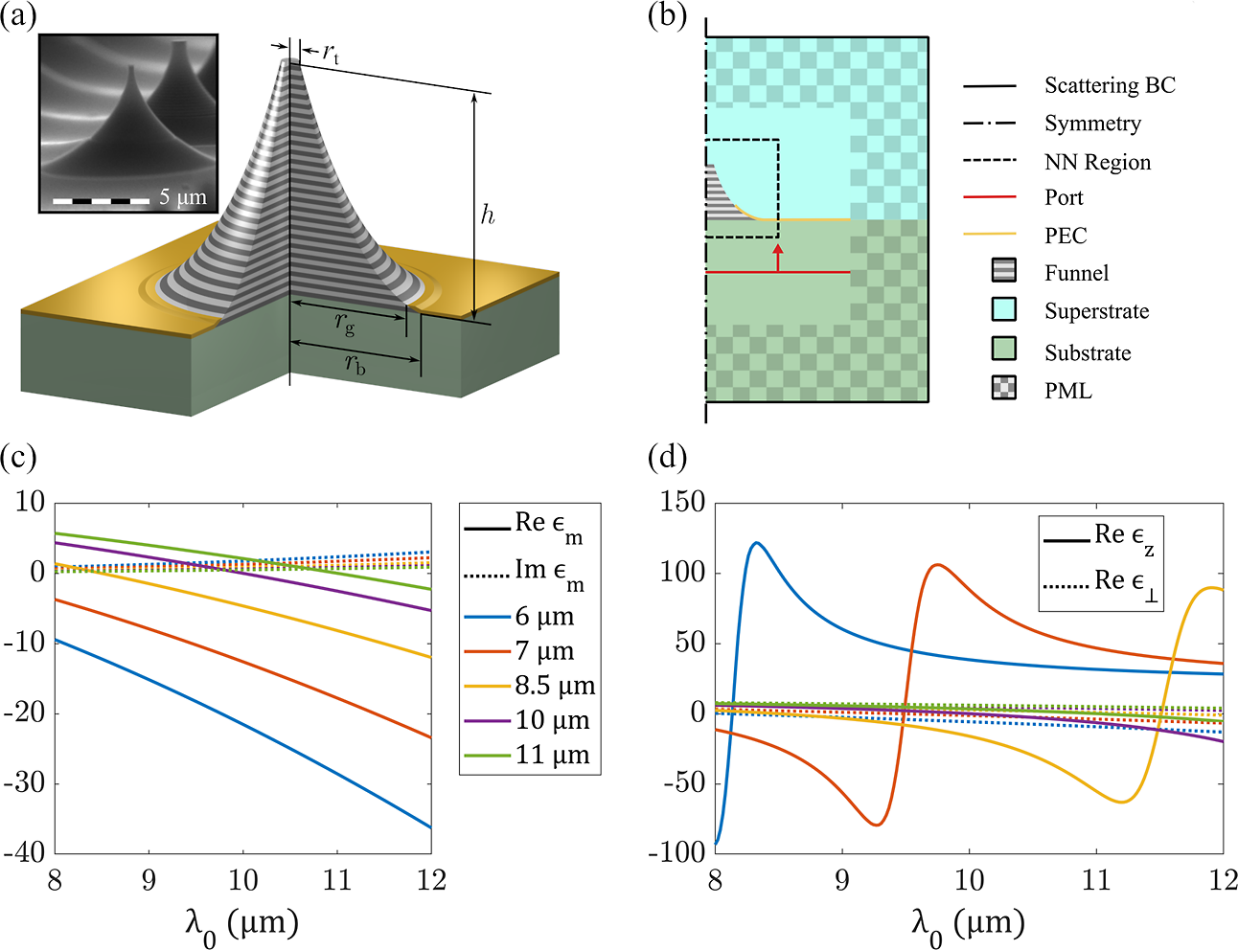
(a) Schematic of the photonic funnel with cut-out demonstrating the composite structure of the core; inset shows scanning electron microscopy (SEM) image of the as-fabricated array of funnels. (b) Simulation setup used in FEM-based solutions of Maxwell’s equations; NNs are trained only on a subregion of the FEM data, which contains the funnel; (c,d) wavelength dependence of (c) the permittivity of the highly doped plasmonic components of the funnels and (d) the components of the resulting effective permittivity tensor.

The ordinary waves (which have E⃗⊥ẑ ) satisfy the dispersion relation *k* ^2^ = ϵ_⊥_ω^2^/*c* ^2^, with *k⃗*, ω, and *c* representing the wavevector of the wave, operating angular frequency, and speed of light in vacuum, respectively. This dispersion is identical to that of plane waves propagating in a homogeneous isotropic material with permittivity ϵ_⊥_. On the other hand, the extraordinary, or transverse-magnetic (TM), waves (with *H⃗*⊥*ẑ*) have dispersion $\frac{k_{x}^{2}+k_{y}^{2}}{\epsilon_{zz}}+\frac{k_{z}^{2}}{\epsilon_{⊥}}=\frac{\omega^{2}}{c^{2}}$ . Notably, for anisotropic materials, the dispersion of extraordinary waves is either elliptical or hyperbolic. The topology of the iso-frequency contours strongly depends on the combination of signs of the effective permittivity tensor.

When the components of the permittivity tensor are of opposite signs, the iso-frequency surfaces are hyperboloids. This hyperbolic dispersion has been identified as the enabling mechanism for such unique optical phenomena as negative refraction, strong enhancement of light–matter interaction, and light manipulation in deep subwavelength areas.^2,43–46^ Hyperbolicity can be achieved by alternating layers of dielectric (ϵ_1_ = ϵ_d_ > 0) and plasmonic (ϵ_2_ = ϵ_m_ < 0) layers. In the semiclassical regime (typically, when the layer thickness ≳ 10 nm), the permittivity of the plasmonic layers as a function of angular frequency of light is well described by the Drude model^47^

1 $$\epsilon_{m}\left(\omega \right)=\epsilon_{\infty }\left(1-\frac{\omega_{p}^{2}}{\omega^{2}+i\gamma \omega }\right)$$

with ϵ_∞_, ω_p_, and γ being background permittivity, plasma frequency, and scattering rate, respectively. Here, we use ϵ_∞_ = 12.15 and γ = 10^13^ s^–1^ and parameterize the plasma frequency using the plasma wavelength, λ_p_, via ω_p_ = 2π*c*/λ_p_.

The most common implementation of these metamaterials leverages a 50/50 composition (*d*_1_ = *d*_2_ = *d*). For such systems, topological transitions occur when the permittivity of the plasmonic layers (ϵ_m_) and the weighted permittivity of the mixture (ϵ_⊥_) change signs.^48,49^ The dispersion of TM waves inside the metamaterial is elliptic for shorter wavelengths λ < λ_p_. It changes to type-I hyperbolicity (ϵ_⊥_ > 0, ϵ_*zz*_ < 0) for $\lambda_{p}<\lambda <{\mathrm{\lambda ̃}}_{p}$ , with the renormalized plasma frequency, ${\mathrm{\lambda ̃}}_{p}$ , defined as $Re\left(\epsilon_{⊥}\left({\mathrm{\lambda ̃}}_{p}\right)\right)=0$ . Finally, the dispersion of TM waves in the composite becomes type-II hyperbolic (ϵ_⊥_ < 0, ϵ_*zz*_ > 0) for ${\mathrm{\lambda ̃}}_{p}<\lambda$ .

Photonic funnels, conical waveguides with hyperbolic metamaterial cores,^40–42^ shown in Figure 1, represent excellent examples of structures capable of manipulating light at a deep subwavelength scale. Recent experimental results^40,42^ demonstrate efficient concentration of mid-infrared light with a vacuum wavelength of ∼10 μm to spatial areas as small as ∼300 nm, 1/30th of the operating wavelength, within an all-semiconductor “designer metal” material platform.^49^ Further analysis relates the field concentration near the funnels’ tips to the absence of the diffraction limit within the hyperbolic material and to the anomalous internal reflection of light from the funnel sidewall, which forms an interface oblique to the optical axis.^42^ Importantly, the optical response of realistic funnels can be engineered at the time of fabrication by controlling the doping of the designer metal layers and thereby adjusting the plasma frequency of these layers. The unusual electromagnetic response, strong field confinement, and significant field inhomogeneities make photonic funnels an ideal platform for testing the performance of ML-driven surrogate solvers of Maxwell’s equations.

## 3 Methods

### 3.1 Data Set Description and Generation

To construct a sufficiently diverse set of labeled configurations, we used FEM to solve for electromagnetic field distributions in photonic funnels with plasmonic layers of different doping concentrations corresponding to plasma wavelengths^50^ of 6 μm, 7 μm, 8.5 μm, 10 μm, and 11 μm. Figure 1 illustrates the wavelength-dependent permittivity of plasmonic layers with various doping concentrations as well as the corresponding effective medium response of the layered metamaterials. Note the drastic changes of effective medium response as a function of both wavelength and doping.

For each doping level, wavelength-dependent permittivity and electromagnetic field distributions have been calculated with a commercial FEM-based solver^12^ (that takes into account that all fields are proportional to exp­(−*i*ϕ), with ϕ being the angular coordinate of the cylindrical reference frame) for free-space wavelengths from 8 to 12 μm with increments of 62.5 nm. The FEM model setup is shown schematically in Figure 1a. Electromagnetic waves that are normally incident on the funnel base are generated by the port boundary condition. Perfectly matched layers^11^ and scattering boundary conditions are used to make the outside boundaries of the simulation region completely transparent to electromagnetic waves, thereby mimicking the surrounding infinite space. The model, which explicitly incorporates 80 nm-thick layers in the funnel cores, is meshed with a resolution of at most 40 nm inside the funnel and 200 nm outside the funnels, with the mesh growth factor set to 1.1 to avoid artifacts related to abrupt changes in mesh size.

For every plasma wavelength and operating frequency, the distribution of electromagnetic fields, along with the distributions of permittivities within a small (5 × 12 μm) region of space containing the funnel (see Figure 1), is interpolated onto a rectangular mesh with resolution 12.5 nm × 10 nm along the *r* and *z* directions, respectively, forming the basis for the data sets used in the study. Note that selecting this internal region of space from the FEM simulations allows us to (i) implicitly incorporate the proper boundary conditions for both incident as well as scattered electromagnetic fields and (ii) avoid the implementation of perfectly matched layers, ports, and scattering conditions within the physics-based constraints used in training our NNs.

The original FEM-generated data has been then resampled into three separate data sets:

- low-resolution data set, 20 × 60 pixels with resolution 250 and 200 nm in *r* and *z* directions, respectively
- medium-resolution data set, 100 × 300 pixels with resolution 50 nm × 40 nm
- high-resolution data set, 200 × 600 pixels with resolution of 25 nm × 20 nm

## 4 Neural Network Architecture

On a fundamental level, approximating solutions of Maxwell’s equations within metamaterials with ML necessitates a neural network to map the operating frequency and the distribution of permittivity across the composite to the distribution of electromagnetic fields, a problem that is similar to image transformation. Previous analysis^51,52^ has demonstrated that convolutional neural networks (CNNs) excel in image transformation. Specifically, encoder-decoder, CNN, and *U*-net architectures have shown success in electromagnetic problems,^19,21,53–55^ presumably due to the cores of the networks learning some low-dimensional representation of the solutions.^34,56^ Note, however, that the vast majority of previous ML-driven solvers of Maxwell’s equations^21,39,57,58^ have analyzed dielectric composites (where electromagnetic fields are relatively smooth) and were trained on relatively large data sets.^14,20,39^

We follow the general approach of constructing *U*-nets. The design of our networks is summarized in Figure 2. Starting with the pixel resolution of the data set, the proposed CNNs reduce the dimensionality of the problem to 20 × 10 pixels and then expand the resulting distributions to their original size.

**Figure 2:**
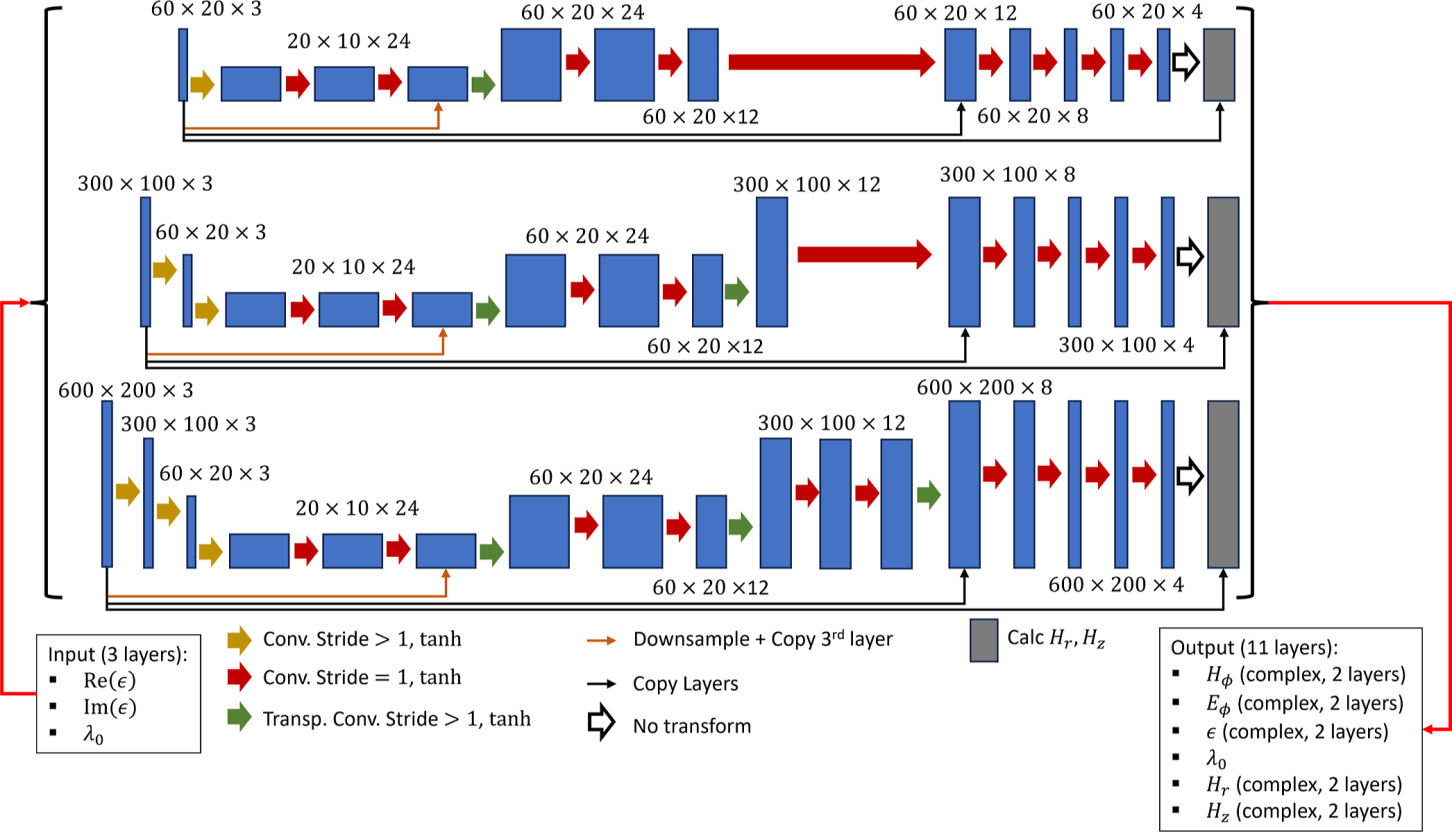
Setup of the CNN used in the study; the three rows represent low-, medium-, and high-resolution networks; boxes represent the size of data as it propagates through the network; arrows represent CNN data operations: each thick solid arrow represents the combination of a (transposed) convolutional layer and a tanh activation layer; thin black and orange arrows represent skip connections; thin red arrows represent input and output.

The linear parts of the network employ standard convolutional and transposed convolutional layers with stride = 1 for those parts of the network that preserve pixel size and with stride >1 for those that perform encoding/downsampling and decoding/upsampling. Hyperbolic tangent activation layers are used to add nonlinearities to the CNN. Combinations of convolutional and tanh layers are marked as thick arrows in Figure 2. In addition, custom layers are introduced to implement skip connections that propagate the vacuum wavelength and permittivity distributions into the depth of the network for both stability of the resulting NN and to enable evaluation of the physics-consistency of the resulting predictions. These layers operate by directly appending several layers of pixels to the output of a given convolutional layer (thin black arrows in Figure 2) or by first downsampling to the core resolution and then concatenating (thin orange arrows in Figure 2).

The base part of the NN (blue layers in Figure 2) is designed to learn the distribution of the ϕ components of the electric and magnetic fields. Note that in our hierarchical setup, the core of the networks remains the same, independent of the resolution of the data set, with the outer structure producing encoding/decoding from/to the higher resolution. The inner structure of the network (layer dimensionality and filter size) was optimized using the low-resolution data set. The medium- and high-resolution networks build upon this geometry by adding “hierarchical” downsampling and upsampling layers, implemented via convolutional and transposed convolutional layers in our NNs. Our analysis suggests that it is important to initialize the downsampling layers with unit weights, thereby setting the network for brute-force averaging of permittivity during the initial training iterations.

The physics-agnostic portion of the CNN, which is trained to produce the ϕ components of the magnetic and electric fields, is followed by a physics-informed layer (gray layers in Figure 2), which calculates distributions of the *r* and *z* components of the magnetic field based on analytical expressions derived from Maxwell’s equations

2 $${\mathrm{E⃗}}_{rz}=\frac{-i}{\epsilon \frac{\omega^{2}}{c^{2}}-\frac{1}{r^{2}}}\left(-\frac{1}{r}{\mathrm{D⃗}}_{rz}E_{\phi }-\frac{\omega }{c}\mathrm{\phi ̂}\times {\mathrm{D⃗}}_{rz}H_{\phi }\right)$$

3 $${\mathrm{H⃗}}_{rz}=\frac{-i}{\epsilon \frac{\omega^{2}}{c^{2}}-\frac{1}{r^{2}}}\left(-\frac{1}{r}{\mathrm{D⃗}}_{rz}H_{\phi }+\epsilon \frac{\omega }{c}\mathrm{\phi ̂}\times {\mathrm{D⃗}}_{rz}E_{\phi }\right)$$

where we have introduced the vector differential operator ${\mathrm{D⃗}}_{rz}f=\mathrm{r̂}\frac{1}{r}\frac{\partial }{\partial r}\left(rf\right)+ẑ\frac{\partial f}{\partial z}$ .

Because the fields are discretized on a regular rectangular grid, all derivatives are approximated with finite difference schemes. Forward and backward differences are used at the edges of the computational domain, while central differences are used within it. Our implementation of the CNNs used in this work and the data sets used in training are available on GitHub and Figshare, respectively.^59,60^

As seen from eq 3, predictions for *H*_*r*_ and *H*_*z*_ may diverge when *ϵr* ^2^ω^2^/*c* ^2^ ≈ 1. This instability is a direct consequence of applying differential operators in a cylindrical geometry. Here, we address the related issues by introducing a regularizing function (see below and Supporting Information). Our approach, illustrated here on an example of cylindrical geometry, may be generalized to other curvilinear coordinates.

### 4.1 Knowledge Transfer between Different NNs

As mentioned above, in the limit of ultrathin layers, the optics of multilayer metamaterials can be adequately described by the effective medium theory. In a related but separate scope, the *U*-shaped NNs are hypothesized to learn low-dimensional representations of the underlying phenomena. These considerations motivate the hierarchical design of the NNs used in this work.

To explore whether the learning outcomes of the NNs are consistent with the effective medium description, we performed a series of experiments where pretrained lower-resolution networks were used as pretrained cores of higher-resolution transfer-learning (TL) networks. In these studies, the learning parameters of the pretrained “core” layers were frozen, with only the averaging and transposed convolution peripheral layers of the higher-resolution NN being trained.

At the implementation level, we drew inspiration from the ResNet^61^ architecture’s approach of organizing layers into “residual blocks.” Specifically, we grouped the frozen layers into a single block, with the internal layer weights corresponding to those of the selected pretrained network. The forward function was designed to perform training within the layers of the block; however, during back-propagation, the weight updates bypass the internal layers of the block, passing directly to the previous layer.

We explored knowledge transfer from low- to medium-resolution networks as well as from medium- to high-resolution networks.

### 4.2 Training Protocols

To assess the benefits of the physics-based constraints, three different regimes of training the CNN are explored. In the base-case black-box (BB) scenario, the model minimizes only the radially weighted mean-squared error of the ϕ components of the electric and magnetic fields (directly produced by the physics-agnostic part of the network) $L_{\phi }=\left\langle w\left(r\right)\left[{\left|H_{\phi }^{Y}-H_{\phi }^{T}\right|}^{2}+{\left|E_{\phi }^{Y}-E_{\phi }^{T}\right|}^{2}\right]\right\rangle$ . Here, the superscripts *Y* and *T* correspond to the predicted and ground-truth fields, respectively, the angled brackets, ⟨···⟩, represent an arithmetic mean over the simulation region, and the radial weight function, *w*(*r*), is used to emphasize the region of small radii where the funnel is located.

The second, field-enhanced (FE) model utilizes a hybrid loss that combines the above-described *L*_ϕ_ with its analog for the remaining components of the magnetic field

4 $$L_{FE}=L_{\phi }+L_{rz}$$

with $L_{rz}=\left\langle w\left(r\right){\left|R\right|}^{2}\left[{\left|H_{r}^{Y}-H_{r}^{T}\right|}^{2}+{\left|H_{z}^{Y}-H_{z}^{T}\right|}^{2}\right]\right\rangle$ and the *rz* components of the magnetic field being produced by the physics layer of the CNN.

In order to prevent the instability of eq 3 from dominating the overall loss, we introduce the regularization function, *R*(*r*, *z*), such that *R*(*r*, *z*) → 0 when $r\to c/\left(\sqrt{\epsilon \left(r,z\right)}\omega \right)$ (see the Supporting Information for details). Because calculation of the *r* and *z* field components requires differentiating the ϕ components, the addition of *L*_*rz*_ allows the CNN to learn the relationships between the spatial field distributions and the distributions of their derivatives. Importantly, evaluation of both *L*_ϕ_ and *L*_*rz*_ terms requires the training set to contain the solutions of Maxwell’s equations (labeled data).

Finally, physics-guided (PG) training combines the above labeled-data-dependent terms, *L*_ϕ_ and *L*_*rz*_, with the physics loss

5 $$L_{ph}=\frac{1}{\max\left|H_{\phi }^{Y}\right|}\left\langle \left|\frac{\partial \left(H_{z}^{Y}R^{2}\right)}{\partial r}-\frac{\partial \left(H_{r}^{Y}R^{2}\right)}{\partial z}+i\frac{\omega }{c}\epsilon E_{\phi }^{Y}R^{2}-2\left(H_{z}^{Y}R\frac{\partial R}{\partial r}-H_{r}^{Y}R\frac{\partial R}{\partial z}\right)\right|\right\rangle$$

which represents the (regularized) residual of Maxwell’s equations for the *H*_ϕ_ component of the field (see the Supporting Information). Therefore, PG training aims to enforce consistency of the solutions that are generated by the NN with Maxwell’s equations. Notably, an evaluation of the physics loss does not require labeled data. As a result, unlabeled-trained (UL) networks can utilize a combination of labeled and unlabeled data, with the former inherently incorporating the boundary conditions and the latter allowing the expansion of the training set without computing additional PDE solutions. This UL loss was also used in training the TL networks described in the preceding section.

Previous analysis^6^ demonstrated that BB- and PG-loss often compete with each other. Here, this competition reflects the different differentiation schemes used by FEM and the PG-loss as well as the existence of multiple solutions to Maxwell’s equations (for example, the trivial solution E⃗=H⃗=0 ) that do not necessarily satisfy the boundary conditions that are implicitly enforced by labeled data. To guide the network toward the correct implementation of boundary conditions, the weight of the physics-loss, *w*_ph_, is dynamically adjusted during training,^6^ resulting in the dynamic PG loss

6 $$L_{PG}=L_{\phi }+L_{rz}+w_{ph}L_{ph}$$

In order to assess the ability of the networks to interpolate and extrapolate between data sets having plasmonic layers with different plasma wavelengths, we train the networks on 50% of the data with plasma wavelengths of 6 and 11 μm or with plasma wavelengths of 7 and 10 μm and add up to 10% of the labeled data from other data sets to the training. The UL models are also provided configurations from the remaining data sets as unlabeled data. The training scenarios are summarized in Table 1, which gives the percent of each data set that was used as labeled and unlabeled data in each network type. Each training scenario has been used to train at least 10 different networks of each resolution and loss type, with the dynamics of their training and validation loss presented in the Supporting Information and their averaged performance summarized below.

**Table 1:** Labeled and Unlabeled Training Data Composition of Each Network

	labeled %					unlabeled %				
	λ_p_ (μm)					λ_p_ (μm)				
network	6	7	8.5	10	11	6	7	8.5	10	11
BB_*i*_, FE_*i*_, PG_*i*_	50	0	10	0	50	none				
TL_*i*_, UL_*i*_	50	0	10	0	50	0	0	40	0	0
BB_*e*_, FE_*e*_, PG_*e*_	0	50	10	50	0	none				
UL_*e*_	0	50	10	50	0	25	0	40	0	25
BB_*x*_, FE_*x*_, PG_*x*_	10	50	10	50	10	none				
UL_*x*_	10	50	10	50	10	25	0	40	0	25

## 5 Results

To demonstrate the impact of physics-based constraints on the accuracy and consistency of NN-predicted fields, we analyze the dependence of the three average losses introduced above (*L*_ϕ_, *L*_*rz*_, and *L*_ph_) both on the enforcement of physics-consistency and on the presence of unlabeled data during training. Sample field distributions are presented to illustrate the models’ performance. Finally, we analyze the generalizability of the models by evaluating their performance across the plasma wavelengths of the plasmonic layers.

The three components of the loss, *L*_ϕ_, *L*_*rz*_ and *L*_ph_, are arranged in increasing degree of physics consistency and simultaneously decreasing reliance on data. Indeed, *L*_ϕ_, which analyzes only the physics-agnostic output of the networks, relies exclusively on data. *L*_*rz*_, which primarily relies on the output of the physics layer, enforces the relationships between the fields at neighboring points [see eq 3]. Lastly, *L*_ph_ exclusively analyzes physics-consistency and pays no regard to data consistency. Our analysis (see below) illustrates that training with *L*_ph_ not only improves the consistency with eq 5 but also improves other metrics that are related to Maxwell’s equations, such as energy conservation—as analyzed through the Poynting theorem (see Supporting Information).

### 5.1 Impact of Physics Information on Accuracy

The performance of the different models is summarized in Figure 3. With the comparatively simple low-resolution model, adding the physics-based layer to the network and adding the *L*_*rz*_ component to the loss function provides enough additional information to adequately represent the coarsely sampled data. Providing the network additional physics-based information (by implementing PG loss) does not quantitatively boost the performance of the model—due to a combination of the model’s simplicity and the mesh being too coarse to resolve the composite structure.

**Figure 3:**
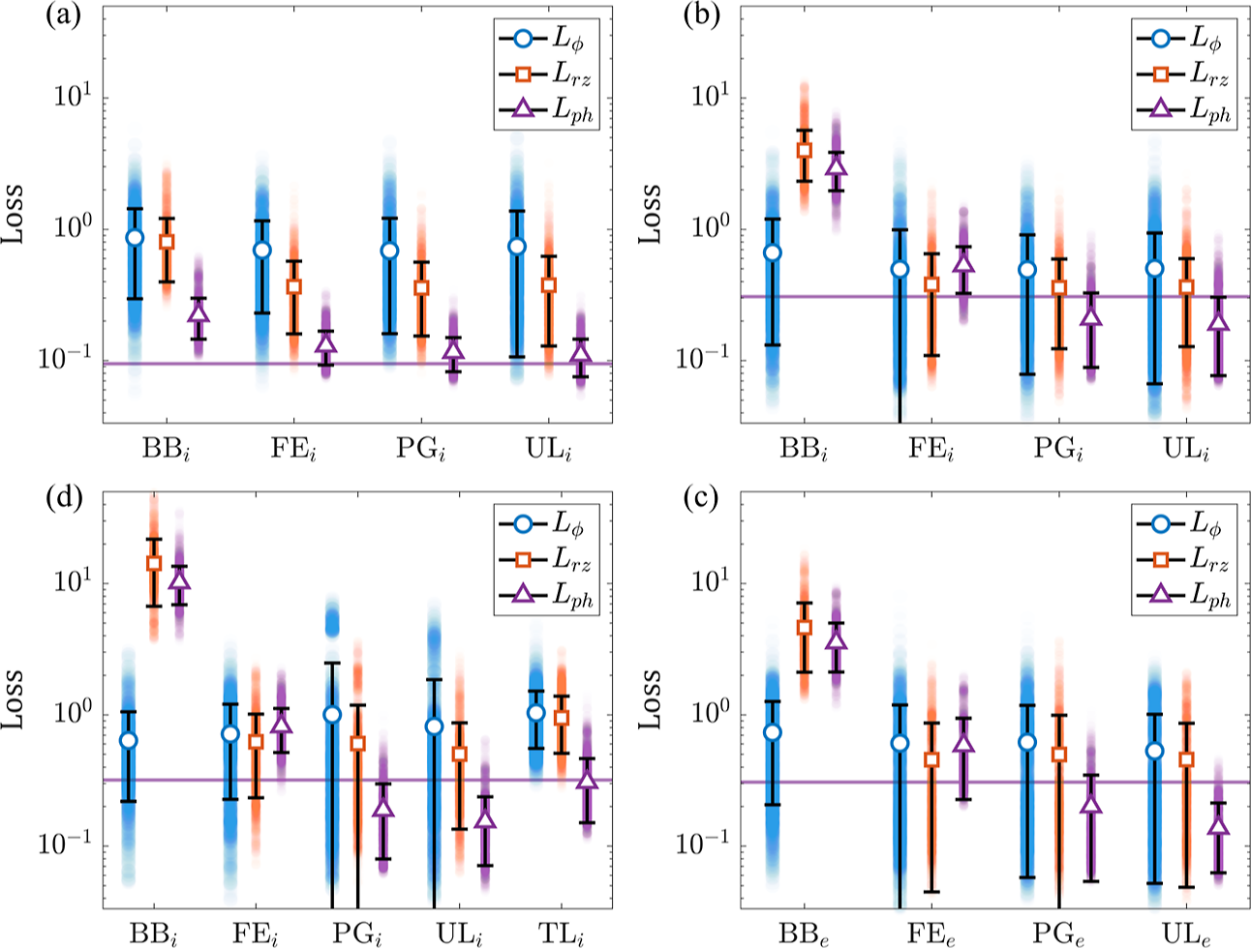
Performance of NNs with different architectures and training protocols, evaluated on the data that was not used in training; panels (a–d) represent low-resolution (a), medium resolution (b,c), and high-resolution (d) networks (see Table 1 for network labels); loss metrics of individual predictions are represented as filled semitransparent circles, resulting in the color-coded distributions; solid white markers and black bars represent the mean and standard deviation of these distributions; the purple horizontal lines show the average *L*_ph_ of all interpolated FEM solutions.

As the resolution and complexity of the model grow, increasing physics-based constraints and adding unlabeled data yield measurable improvements in model performance. Interestingly, the extra physics consistency (as demonstrated by the improving *L*_ph_ metric) sometimes comes at the cost of a small increase of *L*_ϕ_. This apparent contradiction results from the fact that the data used in training was generated by reinterpolating FEM solutions from a triangular mesh to a rectangular mesh. As a result, the “ground truth” does not yield vanishing *L*_ph_. As seen in Figure 3, predictions of the neural net tend to be closer solutions to Maxwell’s equations on the rectangular mesh than the FEM-sourced data.

A more granular look at the NN predictions is shown in Figure 4 where representative examples of model predictions are compared with FEM solutions. Note that in contrast to their BB counterparts, PG networks predict smoother fields and resolve individual layers of the structure.

**Figure 4:**
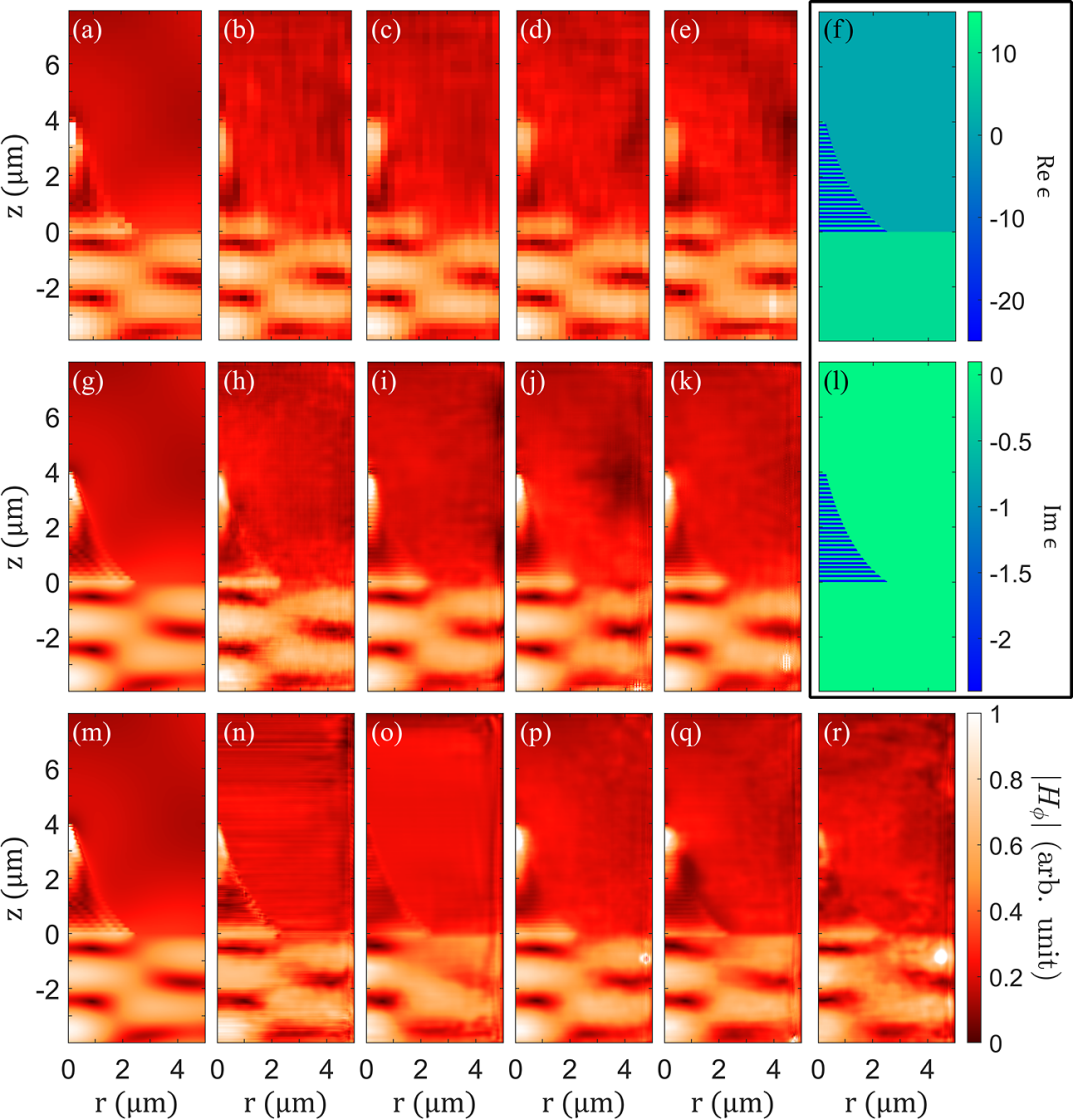
Representative predictions of the NNs with (a–e) low-, (g–k) medium-, and (m–r) high-resolution; input permittivity is shown in panels (f,l); panels (a,g,m) represent ground truth; panels (b,h,n)—predictions of BB_*i*_ NNs, panels (c,i,o)—predictions of FE_*i*_ networks, panels (d,j,p)—predictions of PG_*i*_ networks, and panels (e,k,q)—predictions of UL_*i*_ networks; panel (*r*) illustrates the performance of the TL_*i*_ network. Note that higher-performing networks resolve field oscillations on the scale of individual layers within the composite and field concentration near the tip of the funnel.

Our results are in agreement with previous studies^21,39^ that focused on predictions of field distributions in dielectric structures trained on relatively large (∼10^4^ configurations) data sets. Incorporation of physics loss in these NNs resulted in substantial (but limited) improvements in physics consistency (by a factor of ≲ 2). Here, we see similar dynamics for low-resolution networks that require few labeled-data training inputs to achieve their top performance. At the same time, the physics consistency of our medium- and high-resolution networks, which are trained in the data-poor regime, is improved by an order of magnitude as a result of the incorporation of physics-based constraints.

### 5.2 Knowledge Transfer

As mentioned above, we have attempted knowledge transfer from a pretrained low-resolution network to a medium-resolution network and from a pretrained medium resolution network to its high-resolution counterpart. In both cases, a single average-performing lower-resolution UL network was chosen as the source of the frozen core of the higher-resolution TL networks. Notably, the low-resolution network poorly resolves the individual layers within the composite. Consistent with this design, implementation of PG loss does not substantially improve network performance (see above), and using a pretrained low-resolution network as a learning-free core of the medium-resolution counterpart does not yield adequate performance of the resulting NN.

In contrast, using a pretrained medium resolution network as a (fixed) core of a high-resolution NN provided reasonable performance. As seen in Figures 3 and 4, the accuracy of TL_*i*_ networks falls between the fully trained high-resolution FE_*i*_ and PG_*i*_ NNs.

The physics of finely stratified composites is analytically described by effective medium theories (EMT). In the EMT formalism, the spatial distribution of homogenized (averaged over the scale of the inclusion ∼ *d*) electromagnetic fields is given by effective parameters (here, ϵ_⊥_ and ϵ_*zz*_). These homogenized fields, along with equations that relate the effective medium parameters to microscopic distributions of permittivity, can then be used to recover fine-scale field distributions.^1^

The analytical procedure described above is somewhat similar to the operation of the hierarchical TL CNN reported in this work. Indeed, the CNN-based *U*-nets are known to learn a low-dimensional representation of the underlying phenomena. From this standpoint, while we do not analyze the neural operation of the CNN in detail, the medium-resolution network is likely to learn some form of materials averaging/field recovery by analyzing the transition between the scale of individual layers (resolved at the entrance and exit of the network) and compact representations in its core. The TL high-resolution wraparound parts of the network likely learn the averaging and upscaling procedures. We reserve the analysis of the relationship between the analytical EMT and the operation of TL-based hierarchical CNNs for future work.

By freezing the inner core of the CNN within knowledge transfer networks, we significantly reduce the number of training parameters. Therefore, we expect smaller variability and faster learning in the TL_*i*_ networks as compared with their fully trained high-resolution PG_*i*_ counterparts. However, in our implementation, the time required to calculate one training epoch of a TL_*i*_ network is almost identical to the time required for one epoch of a PG_*i*_ network, indicating that the time spent updating the learnable NN parameters is significantly less than the time spent executing forward and backward propagation steps. Different implementation and optimization settings may affect this result.

At the same time, further analysis (Supporting Information) suggests that TL_*i*_ networks converge over a smaller number of epochs than their PG_*i*_ counterparts. In addition, in our studies, variation between the performance of the best and the worst TL_*i*_ networks was significantly smaller than the variation between the best and the worst PG_*i*_ networks.

### 5.3 Interpolation vs Extrapolation within the Models

As described above (Table 1), the NNs have been trained on multiple subsets of the data derived from FEM solutions, aiming to assess both correctness and generalizability of the proposed PGML networks. Here, we are particularly interested in the ability of the NN to generalize the results between different plasma wavelengths of the doped components of the funnels’ cores.

In the “interpolating” models (subscripted *i*), 50% of the data from the sets representing the lowest and the highest plasma frequencies and an additional 10% from the data set representing the central plasma wavelength were used as labeled training data. The unlabeled networks further included 40% of the central plasma wavelength data set as unlabeled data. Therefore, the CNN would have to deduce the behavior of the composites with λ_p_ = 7 and 10 μm. For the “extrapolating” (subscripted *e*) and “extended extrapolating” (subscripted *x*) networks, a similar approach was used, except with the bulk of labeled training data coming from the 7 and 10 μm plasma wavelengths, having the CNN deduce the behavior of the metamaterials with λ_p_ = 6, 11 μm.

Typically, data interpolation is a much simpler problem than data extrapolation. However, this general rule does not hold for our analysis. As seen in Figure 3b,c, the average performance of the two classes of medium-resolution networks is almost identical to each other, indicating that both interpolation and extrapolation tasks (in terms of λ_p_) in our study represent similar difficulties to the NNs.

Figure 5 provides a more in-depth look at this behavior. In general, as characterized by *L*_ϕ_ loss, the networks perform their best in predicting the fields within the metamaterials for the same plasma wavelength that comprises the majority of their labeled training set. Indeed, *L*_ϕ_ is ∼2 times lower for the data that has a plasma wavelength that is well-represented in the training set than for the configurations with plasma wavelengths that contribute few or no instances to the labeled training data. Incorporation of physics-based constraints improves the physics-consistency of the results for all values of λ_p_ by an order of magnitude, indicating that the CNNs learn the general properties of the field distributions but miss the particular boundary conditions that are encoded in the labeled data.

**Figure 5:**
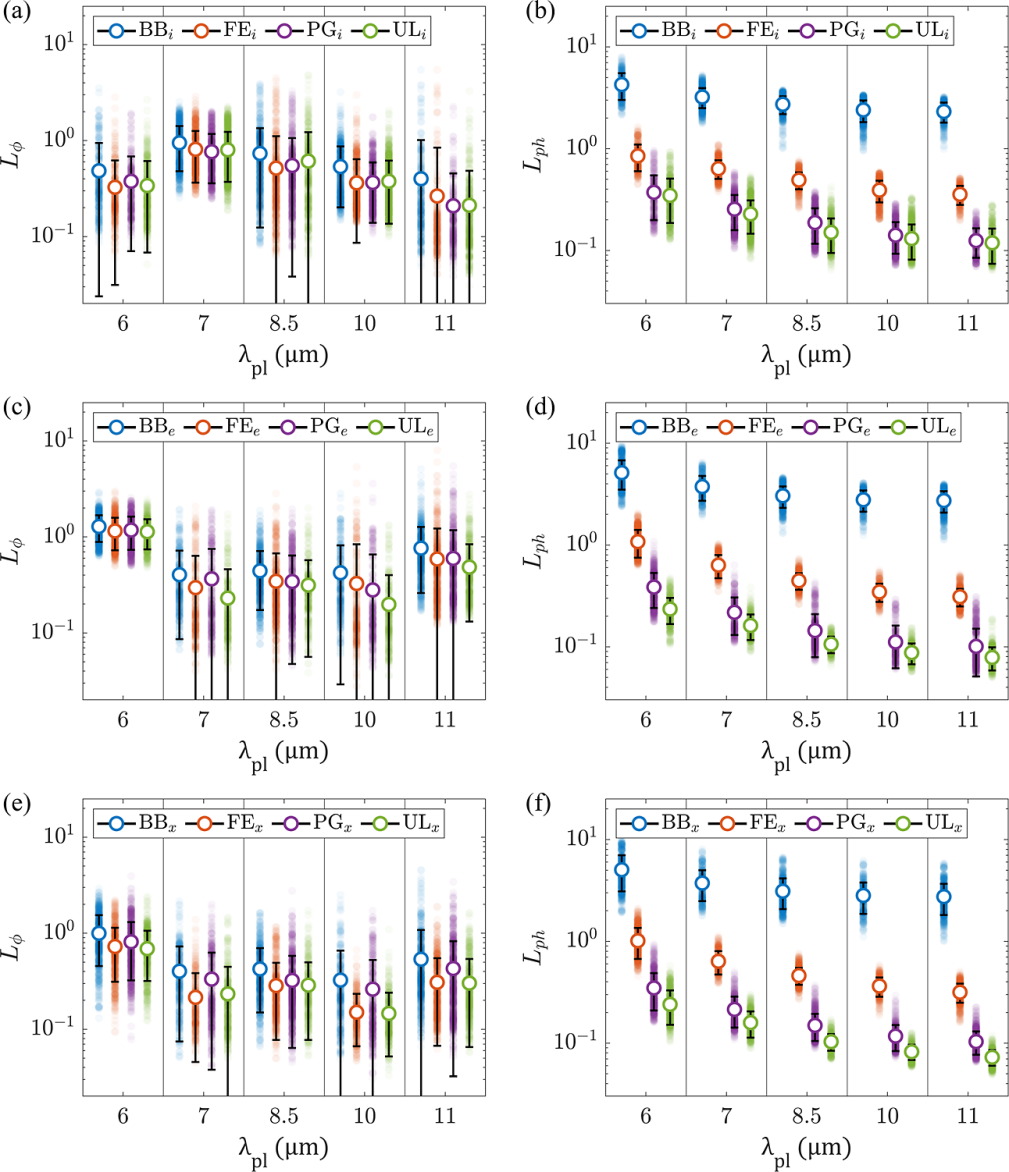
Performance of the medium-resolution networks for predicting the field distribution of composites with given plasma wavelengths; panels (a,c,e) and (b,d,f) represent *L*_ϕ_ and *L*_ph_, respectively, for (a,b) interpolating, (c,d) extrapolating, and (e,f) extended extrapolating networks; colors represent training protocols; individual predictions are represented as filled semitransparent circles, resulting in the color-coded distributions; solid white circle markers and black bars represent the mean and standard deviations of these distributions.

By comparing the performance of extrapolating networks to their “extended” counterparts [Figure 5c,e], it is seen that adding very little labeled data can somewhat address this issue of underspecified boundary conditions: introduction of ∼ 20 labeled distributions (total) for λ_p_ = 6, 11 μm reduces the λ_p_-specific *L*_ϕ_ by ∼20% with almost no effect on *L*_ph_.

Interestingly, in all scenarios, *L*_ph_ decreases as a function of λ_p_. This behavior traces the strength of the resonance in ϵ_*zz*_ that decays and moves out of the spectral range of the study as λ_p_ increases (see Figure 1d).

## 6 Conclusions

We have presented a hierarchical design of PG neural network surrogate solvers of Maxwell’s equations and demonstrated the proposed formalism by predicting the field distributions in hyperbolic metamaterial-based photonic funnels. We have demonstrated that embedding physics information into the ML process, by enforcing the physics-based constraints and by adding unlabeled training configurations, improves the quality of ML predictions in the regime of limited training data. In particular, physics-guided ML predictions are almost 2 orders of magnitude more physics-consistent than their BB–ML counterparts, even near wavelengths where the layered composite undergoes topological transitions. Separately, we have demonstrated that a hierarchical network architecture enables knowledge transfer from existing pretrained models to higher-resolution NN implementations.

The approach presented can be directly applied to the analysis of complex rotationally symmetric electromagnetic systems. The technique can be straightforwardly extended to quasi-2D geometries with inclusions of various sizes and shapes by using the appropriate coordinate-representations of Maxwell’s equations. The formalism can be further extended to 3D geometries, although we anticipate that such extensions will require significantly larger computational resources.

## Supplementary Material



## Notes

The following files are available free of charge. The Supporting Information is available free of charge at https://pubs.acs.org/doi/10.1021/acsphotonics.5c00552.

Analytical derivation of the regularized physics loss *L*_ph_, elaboration on our chosen regularization and radial weight functions, demonstration of the effect of physics consistency on energy conservation, and analysis of the training and validation loss dynamics of our networks (PDF)

## Notes

A preprint of this work is available on arXiv, DOI: 10.48550/arXiv.2502.17644.
