# Supplementary material for: Physics-Guided Hierarchical Neural Networks for Maxwell’s Equations in Plasmonic Metamaterials
Source: ACS Photonics. 2025 Jul 31;12(8):4279–88. doi: 10.1021/acsphotonics.5c00552 (PMC12372168; doi:10.1021/acsphotonics.5c00552)
Supplement: Supplementary file 1 [file ph5c00552_si_001.pdf]

Physics-guided hierarchical neural networks for Maxwell's equations in plasmonic metamaterials; Supplementary Materials

Sean Lynch,[†] Jacob LaMountain,[*,‡] Bo Fan,[‡] Jie Bu,[¶] Amogh Raju,[§] Dan Wasserman,[§] Anuj Karpatne,[¶] and Viktor A. Podolskiy[‡]

†*Miner School of Computer Science*
University of Massachusetts Lowell
Lowell, MA 01854, USA

‡*Department of Physics and Applied Physics*
University of Massachusetts Lowell
Lowell, MA 01854, USA

¶*Department of Computer Science*
Virginia Tech
Blacksburg, VA 24061, USA

§*Department of Electrical and Computer Engineering*
University of Texas Austin
Austin, TX 78712, USA

E-mail: jacob_lamountain@student.uml.edu

Maxwell's Equations in Cylindrical Geometry

As mentioned in the main manuscript, we use the rotational symmetry of our problem to reduce the three-dimensional vectorial Maxwell's equations to equations describing the behavior of the ϕ-components of the electric and magnetic fields (which vary smoothly throughout the geometry). Assuming that all fields are proportional to $\exp(-i\phi)$, once E_ϕ and H_ϕ are known (for example, as predicted by the neural net), the remaining components of the fields can be calculated via

$$\vec{E}_{rz} = \frac{-i}{\epsilon\frac{\omega^2}{c^2} - \frac{1}{r^2}} \left(-\frac{1}{r}\vec{D}_{rz}E_\phi - \frac{\omega}{c}\hat{\phi} \times \vec{D}_{rz}H_\phi \right) \tag{S1}$$

$$\vec{H}_{rz} = \frac{-i}{\epsilon\frac{\omega^2}{c^2} - \frac{1}{r^2}} \left(-\frac{1}{r}\vec{D}_{rz}H_\phi + \epsilon\frac{\omega}{c}\hat{\phi} \times \vec{D}_{rz}E_\phi \right) \tag{S2}$$

where the differential operator $\vec{D}_{rz}$ is defined by $\vec{D}_{rz}f = \hat{r}\frac{1}{r}\frac{\partial}{\partial r}(rf) + \hat{z}\frac{\partial f}{\partial z}$.

Maxwell's equations also provide additional constraints, ensuring self-consistency of the field components:

$$\frac{\partial H_r}{\partial z} - \frac{\partial H_z}{\partial r} = -i\epsilon\frac{\omega}{c}E_\phi \tag{S3}$$

$$\frac{\partial E_r}{\partial z} - \frac{\partial E_z}{\partial r} = i\frac{\omega}{c}H_\phi \tag{S4}$$

the first of which is used as a basis for L_{ph} in the manuscript.

Regularization Function

The neural network directly predicts E_ϕ and H_ϕ, and the physics layer then calculates H_r and H_z using Eq.(S2), above. It is seen, however, that for transparent materials, Eq.(S2) diverges when $r^2\epsilon\omega^2/c^2 = 1$.

In approximate numerical solutions (such as those analyzed in our work), this condition leads to instabilities that – if left unaddressed – would dominate both L_{rz} and L_{ph} loss

functions.

To address these underlying instabilities, we introduce the regularization function $R(r, z)$ in such a way that regularized fields $\vec{\mathcal{E}} = R\vec{E}$ and $\vec{\mathcal{H}} = R\vec{H}$ remain finite within the simulation domain. Explicitly,

$$R(r, z) = 0.1 \frac{\epsilon(r, z) r^2 \frac{\omega^2}{c^2} - 1}{\epsilon(r, z) r^2 \frac{\omega^2}{c^2} + 0.1} \tag{S5}$$

is used in our work (In principle, any $R(r, z)$ that vanishes, at least linearly, when $r^2\epsilon\omega^2/c^2 = 1$ can be used).

To find an appropriate physics loss function, we first recast Eq.(S3) in terms of regularized fields

$$\frac{\partial}{\partial z}\left(\frac{\mathcal{H}_r}{R}\right) - \frac{\partial}{\partial r}\left(\frac{\mathcal{H}_z}{R}\right) = -i\epsilon\frac{\omega}{c}\frac{\mathcal{E}_\phi}{R}. \tag{S6}$$

We then apply the derivatives and rearrange the resulting relationships, arriving at:

$$\frac{\partial}{\partial z}\left(R\mathcal{H}_r\right) - \frac{\partial}{\partial r}\left(R\mathcal{H}_z\right) + 2\left(\mathcal{H}_z\frac{\partial R}{\partial r} - \mathcal{H}_r\frac{\partial R}{\partial z}\right) + i\epsilon\frac{\omega}{c}R\mathcal{E}_\phi = 0. \tag{S7}$$

Recasting the latter equation back to the actual fields yields the physics residual used for physics loss in our work

$$\Lambda_{ph} = \frac{\partial}{\partial z}\left(R^2 H_r\right) - \frac{\partial}{\partial r}\left(R^2 H_z\right) + 2\left(RH_z\frac{\partial R}{\partial r} - RH_r\frac{\partial R}{\partial z}\right) + i\epsilon\frac{\omega}{c}R^2 E_\phi. \tag{S8}$$

Note that physics-consistent solutions should satisfy $\Lambda_{ph}(r, z) \equiv 0$.

Radial Weight Function

The fundamental electromagnetic phenomena enabled by photonic funnels (anomalous reflection and subdiffractive light confinement) are encoded in the field distributions within and in close proximity to the funnels. Since these field distribution features are more important for understanding the electromagnetism of the funnels and at the same time are more com-

plicated than the diffraction-limited field distributions outside the funnels, a weight function is used to improve learning of the fields at small radii. In our networks, this radial weight function is a sigmoid given by

$$w(r) = \frac{5}{1 + 10e^{2(r-3)}} + 0.5, \tag{S9}$$

where r is in µm.

Physics-Consistency and Energy Conservation

An important physical principle, which should hold (at least approximately, given our discretization) for physically consistent fields is energy conservation. We may therefore use the deviation from energy conservation as an additional measure of physics-inconsistency. Furthermore, we can utilize this metric to demonstrate the impact of imposing physics-consistency through the inclusion of L_{ph} in the training loss function.

The conservation of energy for monochromatic fields in linear dispersive media can be written as

$$\frac{\omega}{c}\mathrm{Im}\left\{\epsilon\left|\vec{E}\right|^2 + \mu\left|\vec{H}\right|^2\right\} - \nabla \cdot \mathrm{Re}\{\vec{E} \times \vec{H}^*\} = 0. \tag{S10}$$

The left-hand side of this equation acts as a kind of “Poynting residual” when applied to electromagnetic fields, with nonzero values representing local violations of energy conservation.

As was the case with Λ_{ph}, to make use of this residual with network-predicted fields, we must suppress the numerical instabilities which arise from calculating the r and z field components via Eq.S1 and Eq.S2. Because this residual expression is proportional to the square of the fields, adequately suppressing the numerical errors introduced in calculating these additional components requires multiplication by R^2 resulting in the regularized Poynting

residual:

$$\Lambda_P = R^2 \left(\frac{\omega}{c} \mathrm{Im}\left\{\epsilon\right\} \left|\vec{E}\right|^2 - \nabla \cdot \mathrm{Re}\{\vec{E} \times \vec{H}^*\} \right) \tag{S11}$$

for non-magnetic materials. In analogy with how our physics loss is defined in terms of our physics residual, a Poynting loss, L_P, was calculated as the average magnitude of the Poynting residual normalized by the square of the maximum field, $\max(|E_\phi|, |H_\phi|)$ (here, Gaussian units are used for convenience, making amplitudes of electric and magnetic fields of the plane wave comparable to each other).

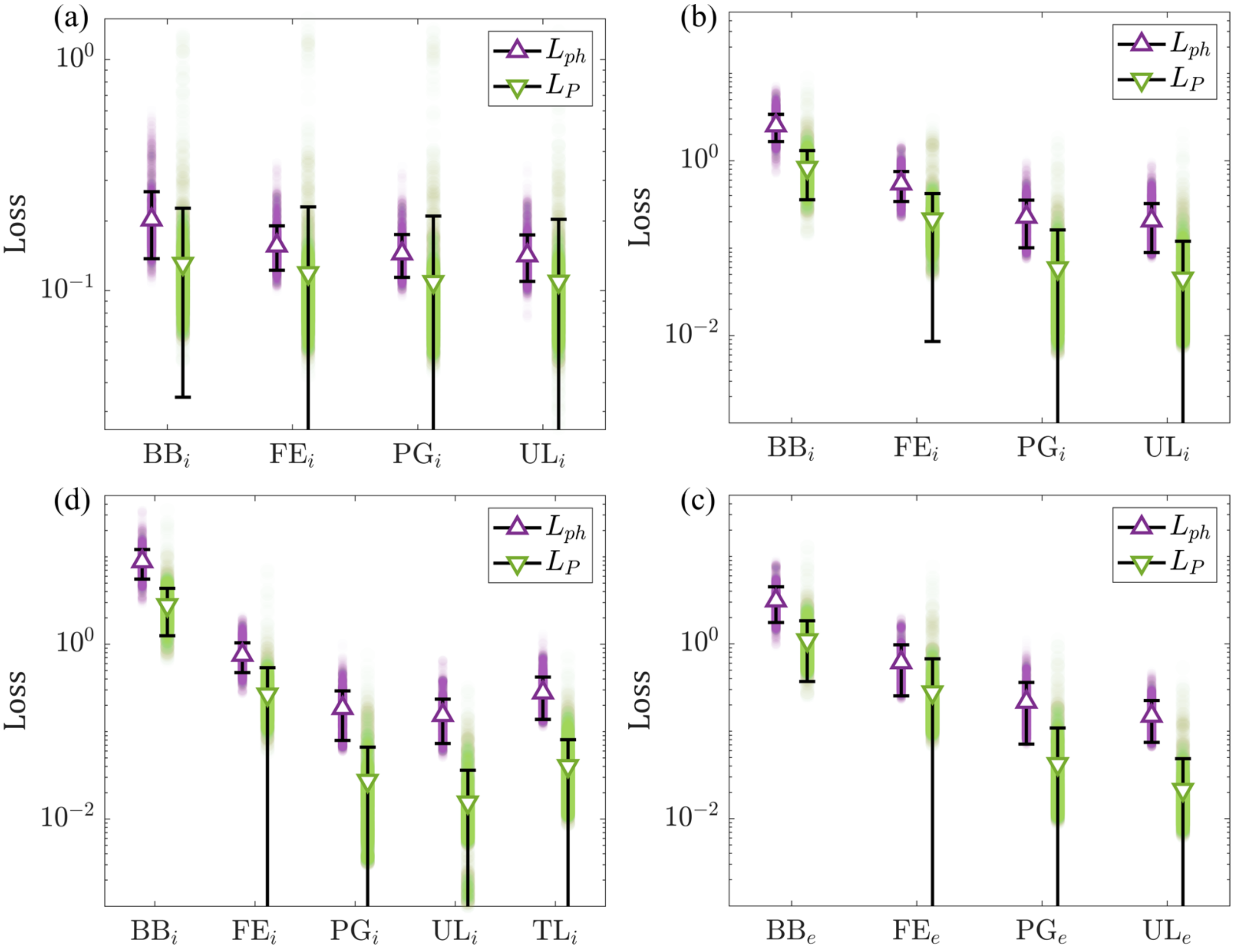

Figure S1: Physics and Poynting losses of NNs with different architectures and training protocols, evaluated on the subset of data that was not used in training sets; panels (a,b,c,d) represent low-resolution (a), medium resolution (b,c), and high-resolution (d) networks; Losses of individual predictions are represented as filled semi-transparent circles; solid white markers and black bars represent the mean and standard deviations of these distributions.

To demonstrate that the physics-consistency enforced by L_{ph} results in fields which better satisfy energy conservation, we calculated the Poynting loss for the predictions of networks of each size across all test data. The results are shown in Fig.S1. It is clearly seen that improving physics-consistency (characterized by lowering L_{ph}) yields improvement in energy conservation (characterized by lowering L_P).

Loss Dynamics

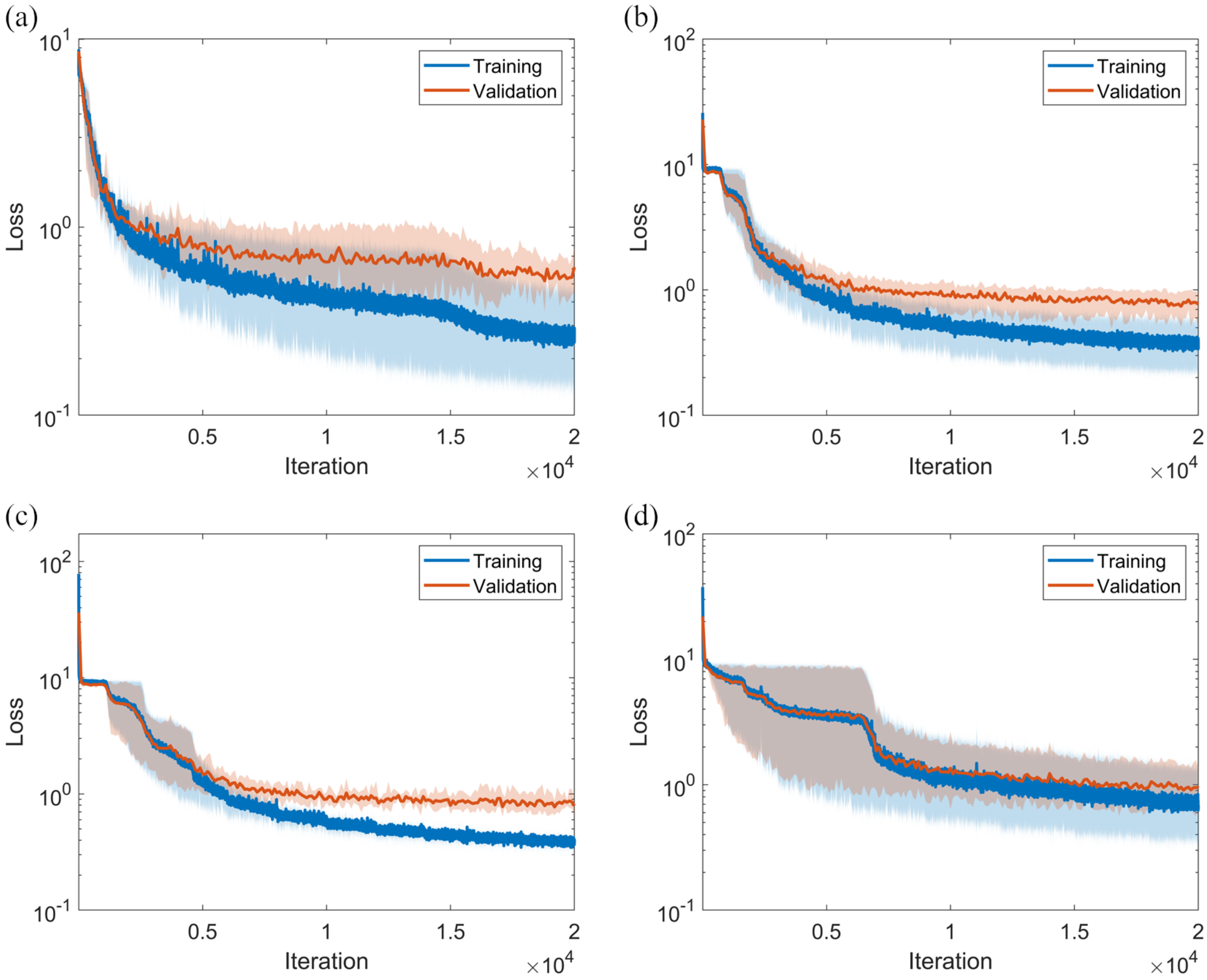

Figure S2: Loss dynamics of medium resolution networks showing training and validation loss against training iteration for (a) black-box, (b) field-enhanced, (c) physics-guided, and (d) unlabeled-trained networks. The solid lines show the losses averaged over networks while the shaded regions are bounded by the maximum and minimum losses at each iteration.

The training and validation loss curves for each of the four medium resolution "interpolating" network configurations are presented in Fig. S2. Across all cases, the validation loss closely follows the trend of the training loss throughout the training process. While the two losses begin at similar values—occasionally with the validation loss slightly lower during the early epochs—they gradually separate as training progresses with the validation loss becoming slightly greater towards convergence. This pattern is expected given the model's exposure to the training data and indicates stable, consistent generalization to unseen data, with no evidence of significant overfitting.

Similar results were seen for high-resolution networks, pointing again to stable training without overfitting. Additionally, we compared the validation loss dynamics between high-resolution UL_i and TL_i networks, summarized in Fig.S3. It can be seen that TL_i networks require fewer training epochs to converge and tend to outperform their UL_i counterparts during early training iterations. However, UL_i networks tend to eventually outperform their TL_i counterparts.

This dynamics reflects the comparatively smaller parameter space of TL networks, which are therefore more readily able to find optimal network configurations and less likely to fall into (and become stuck within) local minima. However, this smaller network dimensionality comes at the price of expressiveness, somewhat limiting the ability of TL networks to fine-tune their predictions.

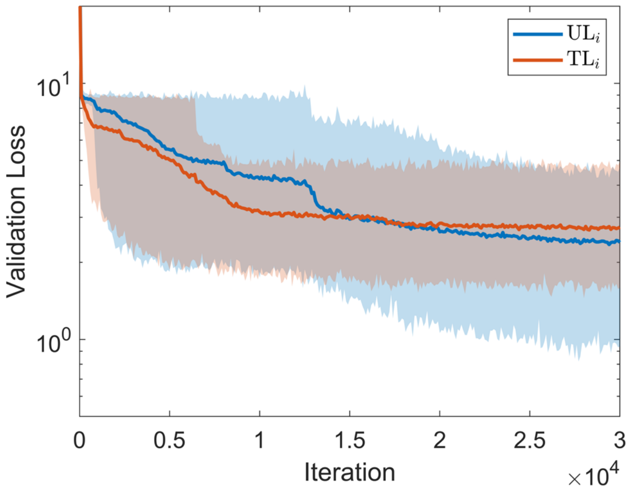

Figure S3: Validation loss dynamics of high-resolution unlabeled-trained and transfer-learning networks. The solid lines show the losses averaged over networks while the shaded regions are bounded by the maximum and minimum losses at each iteration.
